# Variations in oxidative stress and antioxidant defense level during different phases of hibernation in common Asian toad, *Duttaphrynus melanostictus*

**DOI:** 10.1242/bio.058567

**Published:** 2021-08-05

**Authors:** Prabhati Patnaik, Deba Das Sahoo

**Affiliations:** 1 Assistant Scientific Officer, Regional Forensic Science Laboratory, Berhampur, Odisha 760007, India; 2 Post-Graduate Department of Zoology, S.C.S Autonomous College, Puri, Odisha 752001, India

**Keywords:** Hibernation, Oxidative stress markers, Arousal, Super oxide dismutase (SOD), Catalase

## Abstract

To assess redox status during hibernation with metabolic depression, oxidative stress parameters and antioxidant defense were assessed during different phases of hibernation including active period, hibernation, arousal, and post-arousal period, in the liver and brain tissues of *Duttaphrynus melanostictus*. We hypothesized low levels of oxidative stress and antioxidant defense during the hibernation period in comparison to the summer active period, due to hypometabolism and their subsequent increase during the arousal period following an increase in body temperature and metabolism. Contrary to our hypothesis, increased oxidative stress with significantly higher lipid peroxidation, protein carbonylation, oxidized glutathione (GSSG): glutathione (GSH) ratio, and elevated antioxidants defense consisting of higher catalase activity and high ascorbic acid content to control oxidative stress were found during hibernation. However, GSH and uric acid levels were found low with super oxide dismutase (SOD) activities at a steady level during hibernation. Supporting our hypothesis, increased oxidative stress with high lipid peroxidation and GSSG:GSH ratio were found during arousal from hibernation owing to increased oxygen consumption and rewarming. Augmented catalase and SOD activities and nonenzymatic antioxidants (GSH, ascorbic acid, and uric acid) level were found to counteract oxidative stress during arousal periods as it was expected. A steady level of protein carbonylation, indicating no oxidative damage during arousal from hibernation due to elevated antioxidant defense, shows the significance of hibernation to overcome food and water scarcity and cold climatic condition. Decrease in antioxidants levels accompanying coming down of lipid peroxidation, protein carbonylation, and GSSG:GSH ratio to their lower levels during the post-arousal period showing normalcy in redox status as it was during active period indicates controllability of oxidative stress in hibernating toads.

## Abstract (precis)

**Summary:** Hibernation though a period of metabolic depression in common Asian toad, we found oxidative stress and augmented antioxidant defense both during hibernation and arousal from hibernation and coming down of these parameters to basal level during post arousal phase.

## INTRODUCTION

Hibernation is a state of dormancy in response to cold weather and scarcity of food accompanied by low body temperature with many physiological consequences including metabolic depression. In ectothermic animals decrease in ambient temperature (T_a_) and hence reduction in their body temperature (T_b_) induces a decrease in metabolic rate as per the Q_10_ relationship (Withers, 1992; Withers and Cooper, 2010). Though lowering of metabolic rate during hibernation takes place by the thermal Q_10_ effect, some hibernating ectotherms also show an additional intrinsic metabolic depression (Withers and Cooper, 2010). Donohoe and Boutilier (1998) have reported metabolic depression by 40% in *Rana temporaria* staying in water at 3˚C and nearly by 75% during their submerging in hypoxic condition. Reptiles have also shown intrinsic metabolic depression during hibernation, independent of body temperature change (*Phrynosoma m'calli*; Mayhew 1965). This indicates additional intrinsic metabolic depression in response to environmental stressed conditions (hypoxic submerged condition in *Rana temporaria*, hibernation in *Phrynosoma m'calli*) in different animals (Withers and Cooper, 2010).

Metabolic depression during hibernation significantly affects reactive oxygen species (ROS) generation and oxidative stress (Afifi and Alkaladi, 2014). Reduction in oxygen consumption to nearly 20% of normal resting rate and depressed metabolism during hibernation has been reported by Seymour (1973). This reduced oxygen consumption during respiration is likely to result in a decreased production of ROS and lower oxidative stress. However oxidative stress has been reported in endothermic mammals (Carey et al., 2000; Hermes-Lima et al., 2001; Orr et al., 2009) during hibernation. Oxidative stress with increased lipid peroxidation, protein carbonylation, and oxidized glutathione (GSH): reduced GSH (GSSG/GSH) ratio has been reported from hibernating Tibetan frog *Nanorana parkeri* (Yonggang et al., 2018). Several studies in different ectothermic animals have reported increased lipid peroxidation as well as GSSG/GSH ratio indicating oxidative stress during metabolic depression due to both hibernation (Bagnyukova et al., 2003; Yonggang et al., 2018) and aestivation (Carvalho et al., 2010; Storey and Storey, 2012; Jared et al., 2019). These observations indicate that the generation of ROS increases at some point of hibernation/aestivation in Anurans and other ectothermic animals causing oxidative stress. Decrease in aerobic capacity of frog skeletal muscle with low cytochrome-C oxidase, citrate synthase, and lactate dehydrogenase activities have been reported during hibernation of *Rana temporaria* to match the lowered ATP demand during the metabolic depression (St. Pyerri and Boutilier, 2001). Decreased aerobic capacity with reduced oxygen consumption during hibernation/aestivation leads pO_2_ to reach a threshold level in which electrons accumulate at the mitochondrial electron transport chain and causes the generation of superoxide radicals ^−^O_2_^.^ and increased production of H_2_O_2_ (ROS) leading to oxidative stress (Hermes-Lima et al., 2015). Metabolic depression and increased ROS production by isolated mitochondria at moderately lower temperatures have also been reported by Sameh et al., 2010. While the adequate amount of ROS acts as a second messenger for intracellular signaling and regulation, excessive ROS oxidizes biomolecules like lipids, proteins, carbohydrates, and DNA (Kohen and Nyska, 2002). Increased body temperature and oxygen consumption during arousal from hibernation also result in elevated ROS generation and oxidative stress (Hermes-Lima and Zentenosavin, 2002) causing increased lipid peroxidation and redox-sensitive transcription factor (nuclear factor-kB) in endothermic hibernators have been reported by Carey et al., (2000). In response to increased ROS generation and oxidative stress, adequate antioxidant defense comprising both antioxidant enzymes and non-enzymatic antioxidants are required for the maintenance of redox homeostasis during different phases of hibernation. In our previous work (Sahoo and Patnaik, 2020), elevated nonenzymatic antioxidant status during hibernation were reported in the common Asian toad, *Duttaphrynus melanostictus.* Increased oxidative stress and antioxidant activities have also been reported during hibernation in endothermic mammals (Okamoto et al., 2006; Yin et al., 2016; Wei et al., 2018).

The common Asian toad, *Duttaphrynus melanostictus* (Anura: Bufonidae), widely distributed in south and southeast Asia breeds during the rainy season (June to October) and hibernates during the winter months (November to February) (Pratihar and Kundu, 2010; Lin et al., 2011). We found them hibernating inside their burrow of 30±10 cm deep in the moist and red loamy soil of our locality (Paralakhemundi, India; 10°45′ N, 84° 6′ E) or under leaf litter and debris, mostly solitary but sometimes in a group of two to five individuals during the winter months (December and January). Decreased serum thyroxin and decreased O_2_ consumption along with reduced body temperature during hibernation indicating a state of depressed metabolism have been reported in common Asian toads (Pratihar and Kundu, 2009). It corroborates with the finding in aestivating anurans (*Cyclorana* and *Neobatrachus*) by Saymour (1973), Withers (1993), and Withers and Thompson (2000) with respect to decreased oxygen consumption and thyroid activity. Several studies have reported significant depression in aerobic metabolism rate during winter dormancy and aestivation in different anurans (Storey and Storey, 1996; Hudson et al., 2006; Madelaire et al., 2020; Moreira et al., 2020). Unlike some anurans who endure dormancy in a hypoxic microhabitat and undergo anaerobic metabolic depression (Rossi et al., 2020), toads like *Sacphiopus couchii* and frogs like *Pleuroderma diplolistris*, and *Proceratophrys cristiceps* follow aerobic metabolic depression in almost a normoxic microhabitat with air-filled chambers in sandy soil (Seymour, 1973; Flanigan et al., 1990; Cervalho et al., 2010; Moreira et al., 2020). Our observation about microhabitat of hibernating common Asian toads in loose soil with small air chambers that were found indicates normoxic metabolic depression during their hibernation. Terrestrial life, docile behavior, and easy availability in our locality have made the common Asian toad a good model for the study of hibernation physiology.

Though hibernation physiology with respect to oxidative stress and antioxidant defences has been studied in endothermic mammals (Joanisse and Storey, 1996; Hermes-Lima and Storey, 1996; Grundy and Storey, 1998; Holenweg and Reyer, 2000), reports on ectothermic anurans are limited. Repeated cycles of torpor and arousal have been reported during hibernation in endothermic mammals (Storey et al., 2010) with concomitant decrease and an increase in metabolic rate and oxygen consumption. Oxidative stress parameters and antioxidant defense status have also been studied well in their cycles of torpor and arousal. This type of detailed study regarding hibernation in ectothermic animals has not been done. Moreover, most of the studies relating to oxidative stress and antioxidant defense have been done in laboratory conditions simulating hibernation in natural habitats. In the present study oxidative stress parameters and antioxidant defenses in hibernating common Asian toad were examined by collecting them directly from their natural habitat. We hypothesized comparatively low levels of oxidative stress parameters like lipid peroxidation, protein carbonylation, and GSSG/GSH ratio during hibernation due to metabolic depression and subsequent increase in these parameters during arousal from hibernation because of increased oxygen consumption and restoration of metabolic activities. Elevated antioxidant activities are also expected during different phases of hibernation as per the propositions of ‘preparation for oxidative stress (POS)’ (Hermes-Lima et al., 2015; Moreira et al., 2020). Therefore, we examined whether oxidative stress increases in liver and brain tissues of common Asian toads in spite of low oxygen consumption and depressed metabolism during hibernation and what happens to the antioxidant defense status during hibernation. A detailed comparative study of oxidative stress markers comprising lipid peroxidation, protein carbonylation, GSSG/GSH ratio, and antioxidants defense consisting of super oxide dismutase (SOD), catalase, ascorbic acid, uric acid, and reduced glutathione status during the summer active period, hibernation stage, arousal stage, and post arousal stage was done in the present work by taking the animals directly from their natural habitat.

## RESULTS

### Oxidative stress

**Fig. 1.**
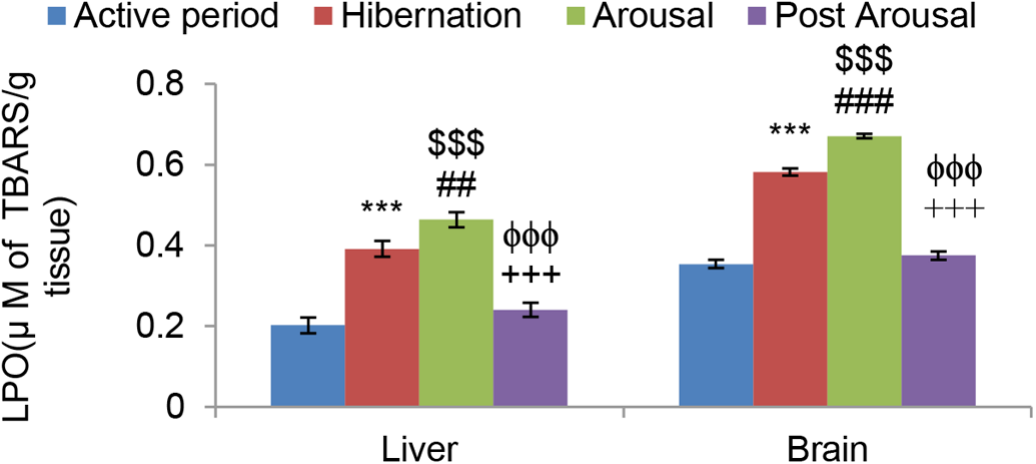
**Effect of hibernation, arousal and post arousal on the level of lipid peroxidation (LPO) of liver and brain tissues of male common Asian toad, *Duttaphrynus melanostictus*.** Data are expressed as the means±s.e.m., (*n*=7). Significant differences calculated using Student's *t*-test from animals during active period are designated as ***(*P*<0.001) compared with active period; ##(*P*<0.01) compared with hibernation;###(*P*<0.001) compared with hibernation; $$$(*P*<0.001) compared with active period; +++(*P*<0.001) compared with arousal; ϕϕϕ(*P*<0.001) compared with hibernation.

**Fig. 2.**
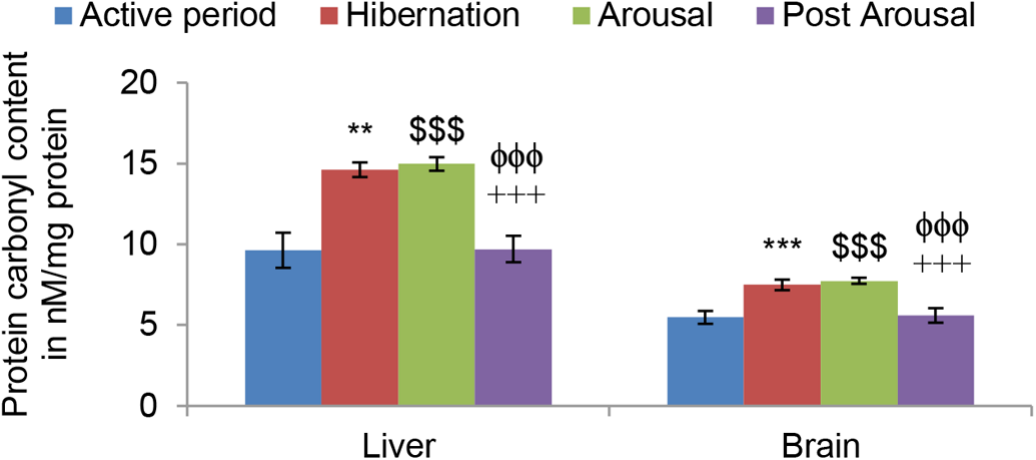
**Effect of hibernation, arousal and post arousal on the level of protein carbonyl content of liver and brain tissues of male common Asian toad, *Duttaphrynus melanostictus*.** Data are expressed as the means±s.e.m, (*n*=7). Significant differences calculated using Student's *t*-test from animals during active period are designated as **(*P*<0.01) compared with active period; ***(*P*<0.001) compared with active period; $$$(*P*<0.001) compared with active period; +++(*P*<0.001) compared with arousal; ϕϕϕ(*P*<0.001) compared with hibernation.

Lipid peroxidation (LPO) level measured in terms of TBARS was found to be significantly higher in both liver (*P*<0.001) and brain (*P*<0.001) tissues during hibernation (Fig. 1) in comparison to tissues from toads during the active period. It also increased significantly during the arousal period in both the tissues in comparison to hibernation and active period. However, during the post arousal period, the LPO level decreased significantly (*P*<0.001), compared to both the hibernation and arousal period in both the tissues, and came down almost to the level that was during the active period. Protein carbonyl content during hibernation was found significantly higher in both liver (*P*<0.01) and brain (*P*<0.001) tissues in comparison to active periods. Unlike lipid peroxidation, there was no significant increase in it during the arousal period, compared with the hibernation period. However, during the post arousal phase, it again decreased significantly (*P*<0.001) compared to the hibernation and arousal period to reach almost the level that was during the active period (Fig. 2).

**Fig. 3.**
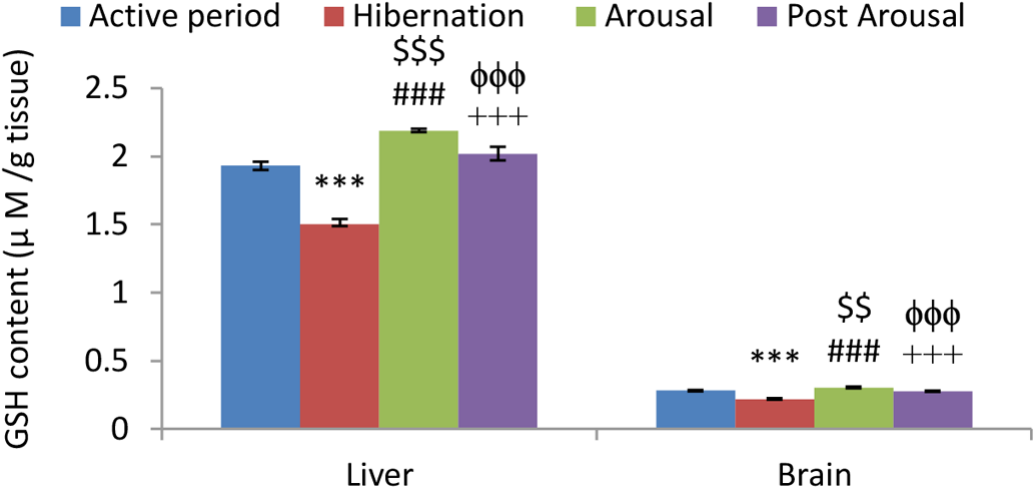
**Effect of hibernation, arousal and post arousal on the level of reduced glutathione content of liver and brain tissues of male common Asian toad, *Duttaphrynus melanostictus*.** Data are expressed as the means±s.e.m., (*n*=7). Significant differences calculated using Student's *t*-test from animals during active period are designated as ***(*P*<0.001) compared with active period; ###(*P*<0.001) compared with hibernation; $$(*P*<0.01) compared with active period; $$$(*P*<0.001) compared with active period; +++(*P*<0.001) compared with arousal; ϕϕϕ(*P*<0.001) compared with hibernation.

**Fig. 4.**
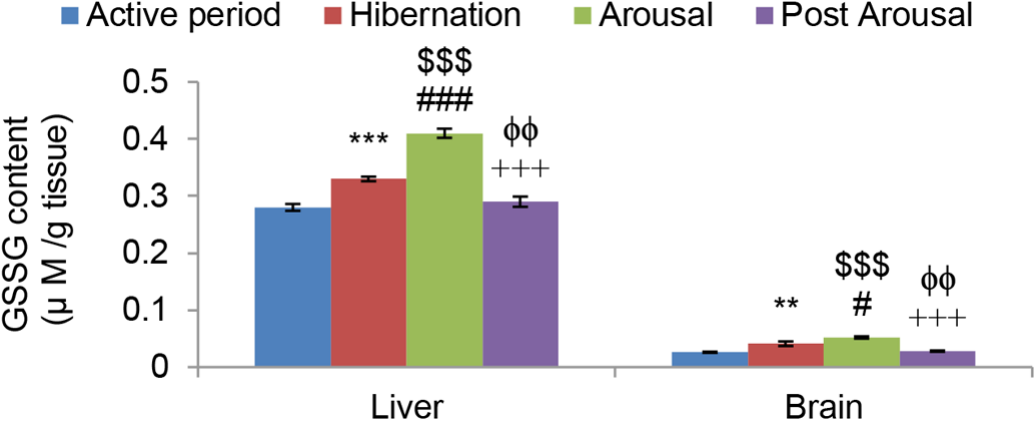
**Effect of hibernation, arousal and post arousal on the level of oxidized glutathione content of liver and brain tissues of male common Asian toad, *Duttaphrynus melanostictus*.** Data are expressed as the means±s.e.m., (*n*=7). Significant differences calculated using Student's *t*-test from animals during active period are designated as **(*P*<0.01) compared with active period; ***(*P*<0.001) compared with active period; #(*P*<0.05) compared with hibernation; ###(*P*<0.001) compared with hibernation; $$$(*P*<0.001) compared with active period; +++(*P*<0.001) compared with arousal; ϕϕ(*P*<0.01) compared with hibernation.

Reduced GSH level was found significantly low in both liver (*P*<0.001) and brain (*P*<0.001) tissues of hibernating toads in comparison to toads during the active period (Fig. 3). GSSG level was found significantly increased in both liver (*P*<0.001) and brain (*P*<0.01) tissues of hibernating toads, compared to active toads (Fig. 4). However, there was a significant decrease in glutathione equivalent (GSHeq=GSH+2GSSG) level during hibernation in both liver (*P*<0.001) and brain (*P*<0.05) tissues compared to active toads in spite of an increase in GSSG level. This decrease in GSHeq during hibernation was due to a decrease in GSH level which led to a significant (*P*<0.001) increase in GSSG:GSH ratio in both liver and brain tissues indicating oxidative stress. Unlike hibernation, there was a significant increase in GSH and GSSG during the arousal phase in both liver (*P*<0.001) and brain (*P*<0.001) tissues in comparison to hibernation and active periods. This also led to a significant increase in GSHeq during the arousal phase in both the tissues (*P*<0.001) compared to hibernation (Table 1). A significant increase in GSH during arousal in both the tissues caused no further significant increase in GSSG:GSH ratio during the arousal phase in comparison to hibernation. However, there was a significant (*P*<0.001) increase in GSSG:GSH ratio indicating oxidative stress during the arousal period in comparison to active periods. During the post arousal period GSH, GSSG, and GSHeq levels decreased significantly (*P*<0.001) in both the tissues compared to the hibernation and arousal period and reached the level that was present during the active period. This caused no significant difference between the GSSG:GSH ratio of the active period and post arousal period showing no oxidative stress.

**Table 1.**
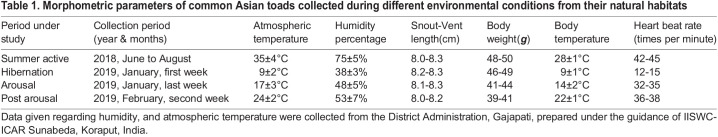
Glutathione status in liver and brain tissues of the common Asian toad, Duttaphrynus melanostictus

### Antioxidant defense

**Fig. 5.**
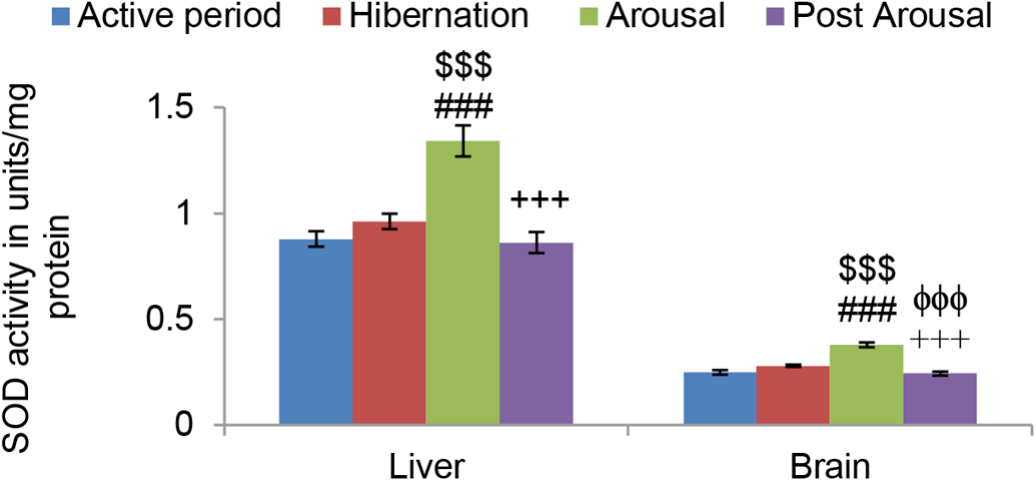
**Effect of hibernation, arousal and post arousal on the level of superoxide dismutase activity of liver and brain tissues of male common Asian toad, *Duttaphrynus melanostictus*.** Data are expressed as the means±s.e.m, (*n*=7). Significant differences calculated using Student's *t*-test from animals during the active period are designated as ###(*P*<0.001) compared with hibernation; $$$(*P*<0.001) compared with the active period; +++(*P*<0.001)compared with arousal; ϕϕϕ(*P*<0.001) compared with hibernation.

**Fig. 6.**
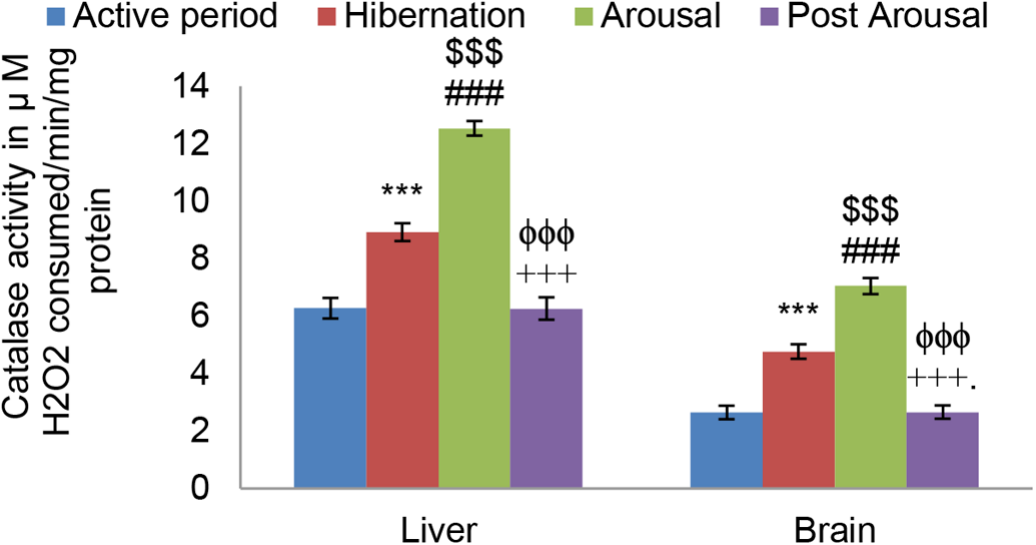
**Effect of hibernation, arousal and post arousal on the level of catalase activity of liver and brain tissues of male common Asian toad, *Duttaphrynus melanostictus*.** Data are expressed as the means±s.e.m, (*n*=7). Significant differences calculated using Student's *t*-test from animals during active period are designated as ***(*P*<0.001) compared with active period; ###(*P*<0.001) compared with hibernation; $$$(*P*<0.001) compared with active period; +++(*P*<0.001) compared with arousal; ϕϕϕ(*P*<0.001) compared with hibernation.

Antioxidant defense comprising antioxidant enzymes (Superoxide dismutase and Catalase) and nonenzymatic antioxidants (Ascorbic acid, Uric acid, and GSH) were investigated in this study. Although there was an increasing trend in SOD activity in both liver and brain tissues of hibernating toads compared to active toads it was found nonsignificant (Fig. 5). However, during the arousal period, SOD activity increased significantly (*P*<0.001) in both the liver and brain tissues in comparison to both hibernating and active period. During post arousal, period SOD activity decreased significantly (*P*<0.001) compared to hibernating and arousal period and almost reached the level that was in the active period. Unlike SOD, Catalase activity increased significantly (*P*<0.001) in both liver and brain tissue during the hibernation period in comparison to the active period (Fig. 6). During the arousal period, there was also a further significant (*P*<0.001) increase in its activity in both the tissues, compared to hibernation and active period. However, it decreased significantly (*P*<0.001) in comparison to the hibernation and arousal period and reached the level that was present during active periods.

**Fig. 7.**
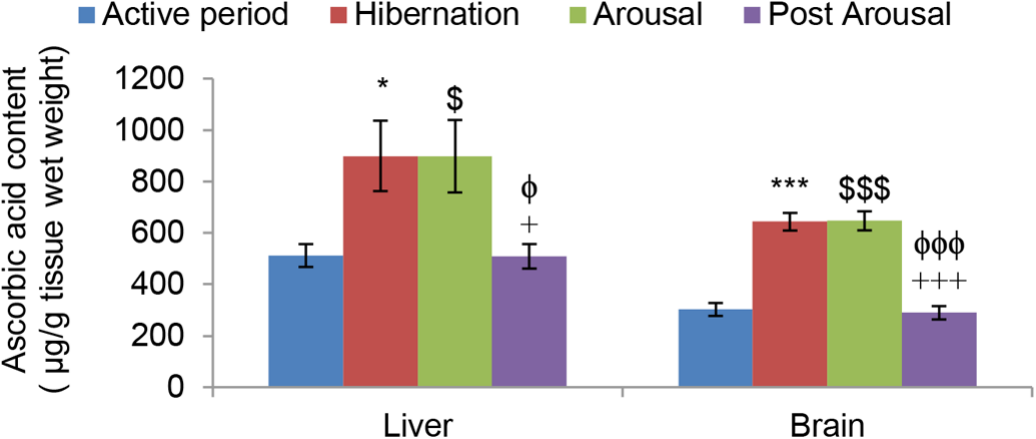
**Effect of hibernation, arousal and post arousal on the level of ascorbic acid content of liver and brain tissues of male common Asian toad, *Duttaphrynus melanostictus*.** Data are expressed as the means±s.e.m., (*n*=7). Significant differences calculated using Student's *t*-test from animals during active period are designated as *(*P*<0.05) compared with active period; ***(*P*<0.001) compared with active period; $(*P*<0.05) compared with active period; $$$(*P*<0.001) compared with active period;+(*P*<0.05) compared with arousal; +++(*P*<0.001) compared with arousal; ϕ(*P*<0.05) compared with hibernation; ϕϕϕ(*P*<0.001) compared with hibernation.

**Fig. 8.**
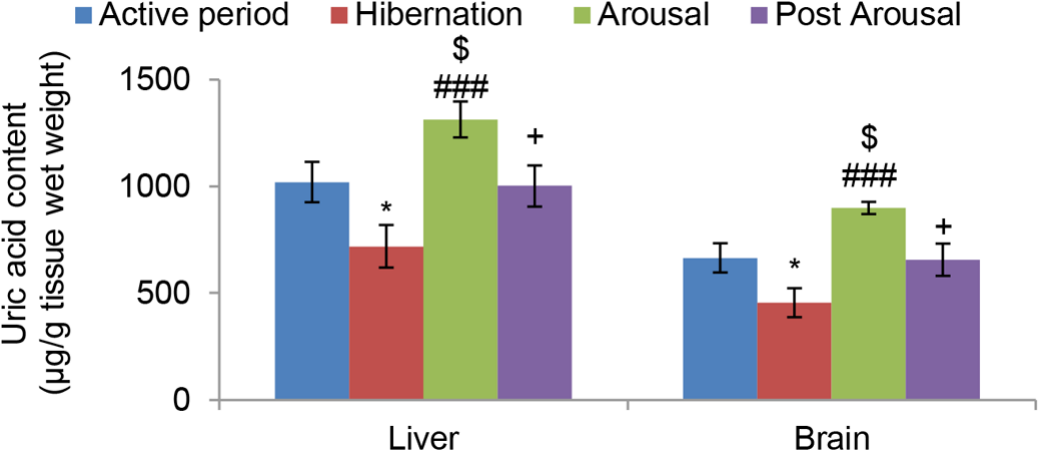
**Effect of hibernation, arousal and post arousal on the level of uric acid content of liver and brain tissues of male common Asian toad, *Duttaphrynus melanostictus*.** Data are expressed as the means±s.e.m, (*n*=7). Significant differences calculated using Student's *t*-test from animals during the active period are designated as *(*P*<0.05) compared with the active period; ###(*P*<0.001) compared with hibernation; $(*P*<0.05) compared with the active period;+(*P*<0.05) compared with arousal.

Nonenzymatic antioxidants small molecules like ascorbic acid increased significantly in the liver (*P*<0.05) and brain (*P*<0.001) tissues during hibernation in comparison to the active period (Fig. 7). However, during the arousal period there was no further significant change in its level in both the tissues, and during the post arousal period it decreased significantly in the liver (*P*<0.05) and brain (*P*<0.001) tissues compared to hibernating and arousal period and reached the level that was in the active period. Unlike ascorbic acid uric acid decreased significantly (*P*<0.05) in both liver and brain tissues during hibernation, compared to the active period (Fig. 8). It then increased significantly (*P*<0.001) in both the tissues during the arousal period in comparison to the hibernation period and decreased significantly (*P*<0.05) during the post arousal period compared to the arousal period and reached the level that was in the active period.

## DISCUSSION

In this study oxidative stress and antioxidant defense comprising both enzymatic and nonenzymatic antioxidant small molecules were investigated in the liver and brain tissues of hibernating common Asian toads. Hibernation characterized by low body temperature and hypometabolism (Withers and Cooper, 2010; Pratihar and Kundu, 2010; Lin et al., 2011) has a potent effect on ROS generation and oxidative stress (Afifi and Alkaladi, 2014). We found a common Asian toad inside their burrow in loose moist loamy soil with small air spaces among the soil peds which indicates normoxic microhabitat. However, to overcome stressful conditions like cold, scarcity of food and water, the animals have undergone a hypometabolic state with low body temperature and heartbeat rate observed by us and reduced thyroxin level and oxygen consumption as reported by Pratihar and Kundu (2009). Contrary to the hypothesis our investigation showed a significantly higher level of lipid peroxidation in terms of TBARS and protein carbonylation in both the tissues studied during hibernation in comparison to the active period. Hibernating toads with reduced oxygen consumption probably produced a considerable amount of ROS that might have caused increased lipid peroxidation and protein carbonylation. Reduced oxygen consumption has been reported to maintain the redox state of the mitochondrial electron transport system towards a reduced state causing the production of superoxide radicals (Hernansanz-Agustin et al., 2014) and thus favoring cellular antioxidant production (Smith et al., 2017). Our observation relating increased lipid peroxidation and protein carbonylation during the early part of the hibernation (≤10 days) is in good agreement with the observation of oxidative stress during preparation for hibernation in the intestine of tegu lizards by Moreira et al., (2018). It also corroborates with the findings of Yonggang et al., (2018) regarding oxidative stress during hibernation in *Nanorana parkeri*. The augmented lipid peroxidation indicates oxidative stress due to increased peroxidation by ROS and decreased lipid peroxide scavenging activity and accumulation of peroxidized lipids that are not replenished by dietary intake and biosynthesis during hibernation with hypometabolism. The increased protein carbonylation indicating oxidative stress may be due to elevated lipid peroxides (Uchida and Stadtman, 1993) and low protease activities for removing carbonylated proteins (Wolff and Dean, 1986) during hibernation. It has also been reported (He et al., 2015) that cold exposure increases polyunsaturated fatty acids (PUFA) in membranes for maintaining its fluidity. Low environmental temperature and corresponding low body temperature could have increased PUFA content in membranes causing them more susceptible to lipid peroxidation. Increased PUFA in the body fat of heterothermic mammals has also been reported before their entry into torpor (Munro and Thomas, 2004). Our findings regarding oxidative stress during hibernation corroborate with the results obtained in different ectothermic (Grundy and Storey, 1998; Yonggang et al., 2018) and endothermic animals (Carey et al., 2000, 2003; Emre et al., 2014).

In support of our hypothesis, we found a significant increase in lipid peroxidation in terms of TBARS in both liver and brain tissues during arousal. Towards the later part of the winter season, with raised atmospheric temperature, the body temperature of hibernating common Asian toad was found to be increasing along with an increase in heartbeat rate, and they were found coming out from their burrows for foraging. These ‘aroused’ toads with rewarming and raised metabolism have increased ROS production (Bagnyukova et al., 2003) and consequently increased lipid peroxidation. However, there was no significant difference in carbonylated protein content in both the tissues studied in toads during arousal in comparison to hibernating toads. A surge in oxygen consumption along with recovery of physiological functions has been reported during arousal in endothermic mammals (Muleme et al., 2006). Increased ROS production due to increased oxygen consumption (Munro and Thomas, 2004; Gavric et al., 2017) and raised metabolic rate might have caused lipid peroxidation (Perez-Campo et al., 1990; Bagnyukova et al., 2003) in both the metabolically active tissues resulting in oxidative stress. However, maintenance of protein carbonylation at a steady-state level during arousal may be due to increased antioxidant defense as observed in our study which is an adaptive mechanism towards increased production of ROS during hibernation. The elevated antioxidant defense in both the tissues could be a preparatory mechanism to minimize the oxidative damage during hibernation and arousal (Hermes-Lima et al., 2001, 2015; Moreira et al., 2017; Giraud-Billoud et al., 2019) and this might be why hibernation shows no tissue oxidative damage. Data from our investigation supports that the level of oxidative stress increases during hibernation and arousal. Besides a significant increase in lipid peroxidation and protein carbonylation during hibernation and arousal, a significant increase in GSSG level was also found. Though reduced GSH level was found significantly decreasing during hibernation compared to active toads, it increased significantly during the arousal phase in comparison to hibernating and active toads in both the tissues. However, the ratio of GSSG to GSH increased significantly during both hibernation and arousal in comparison to its value at the active period. So a comparatively lower level of GSH and higher GSSG levels in both the tissues studied leading to a significantly higher value of the GSSG:GSH ratio indicates oxidative stress (Shelly, 2013; Sentellas et al., 2014) during hibernation and arousal in comparison to active period. Our investigation showed a significant decrease in lipid peroxidation, protein carbonylation level, and GSSG:GSH ratio during the post arousal phase in comparison to the hibernation and arousal phase and reached the level of active period value in both the tissues studied showing normalcy in redox status.

In accordance with our expectation, we found elevated antioxidant defense showing an adaptive response towards oxidative stress. A significant increase in catalase activity in both liver and brain tissues of hibernating toads was found in comparison to the active toads. Further increase in catalase activity was also found during arousal to counteract the possible damaging effects of ROS generated due to increased oxygen consumption and rewarming. However, catalase activity came down during the post arousal phase almost to the level that was in the active period indicating normalcy. It has been reported earlier about the positively correlated ROS generation with mitochondrial respiration and oxygen consumption (Munro and Thomas, 2004; Gavric et al., 2017). To control increased ROS mediated damage, enzymatic antioxidant defense activity increases (Storey, 1996; Wei et al., 2018) which have also been reported by upregulation of catalase activity in liver tissues during arousal in ectothermic animal *Rana ridibunda* (Bagnyukova et al., 2003) and also in endothermic animals like Daurian ground squirrels (Wei et al., 2018). In our investigation SOD activity was found at a relatively steady level in both hibernating and active periods which significantly increased during the arousal phase to counteract the damaging effect of ROS produced due to increased oxygen consumption and rewarming. SOD catalyzes the dismutation of O_2˙_^−^ into H_2_O_2_ which again acted by catalase to convert into H_2_O and O_2_ (Halliwell and Cross, 1994; Chainy et al., 2016). In the present study up-regulation of both SOD and catalase during arousal in both the tissues studied indicates augmented antioxidant enzyme activity to minimize oxidative stress due to the increased ROS production during elevated oxygen consumption and rewarming. Increased catalase activity during hibernation and arousal in both the tissues studied indicates the role of catalase in maintaining a steady level of H_2_O_2_ for better management of redox homeostasis. The difference in SOD and catalase activities that are found in liver and brain tissues is due to their physiological status during hibernation and their susceptibility towards ROS. Our results are in good agreement with the findings of Buzadzic et al. (1990), Ohta et al., (2006), Okamoto et al. (2006), Yin et al. (2016), Wei et al. (2018) in different endothermic mammalian hibernators and also with the report by Hermes-Lima and Storey (1995) in aestivating pulmonate land snail. Increased antioxidant enzyme activities during hypometabolic hibernation and arousal are in good agreement with, ‘preparation for oxidative stress’ (POS) theory which proposes induction of antioxidant enzymes during hypometabolic condition is a way to prepare animals for oxidative damage that may happen ultimately during re-oxygenation (Hermes-lima et al., 2015; Giraud-Billoud et al., 2019; Moreira et al., 2020).

Like antioxidant enzymes, nonenzymatic antioxidant small molecules also act as free radical traps and protect tissues vulnerable to ROS attack. In this investigation, a significant increase in ascorbic acid content in both liver and brain tissues was found in hibernating toads than the active toads. However, there was no further significant increase in its level during the arousal phase. It was found to maintain an almost steady level throughout the hibernation and arousal phase, suggesting its protective role during hibernation and arousal. During post arousal phase ascorbic acid level decreased to the level that was found in active toads. Ascorbic acid is a well-known antioxidant (Chakrabarty et al., 1992; Ames et al., 1993) in amphibians synthesized in the kidney (Grollman and Lehninger, 1957; Chatterjee, 1973) and distributed to other tissues for transportation into cells by sodium-dependent processes. It has been reported to protect the tissues from the ROS attack during hibernation and arousal (Drew et al., 1999; Toien et al., 2001). The present results showing elevated ascorbic acid content in liver and brain tissues corroborate this. A comparatively higher increase in ascorbic acid level in brain tissues than the liver tissues as observed in this study may be an adaptive response to counteract lipid peroxides that are produced in brain tissues due to its high PUFA content and also to act as a neuroprotectant (Fiona and James, 2009). Unlike ascorbic acid, uric acid is produced locally as a product of purine metabolism catalyzed by xanthine oxidase in response to oxidative stress (Halliwell and Gutteridge, 1988). In the present investigation, significantly lower uric acid content was found in both liver and brain tissues of hibernating toads in comparison to active toads. However uric acid content significantly increased in both the tissues during the arousal phase and decreased to a level almost that was inactive toads during the post arousal phase. A decrease in uric acid content during hibernation may be due to its low rate of synthesis because of low xanthine oxidase activity at low body temperature and hypometabolism. Low uric acid content has been reported in the liver of hibernating ground squirrel due to low AMP deaminase activity (Miguel et al., 2015). Uric acid as a scavenger of free radicals (Davies et al., 1986) and is capable of maintaining ascorbic acid in its reduced state (Sevanian et al., 1985), has increased in both the tissues studied to protect them from increased ROS attack during the arousal phase.

GSH, a water-soluble endogenous antioxidant tripeptide also maintains exogenous antioxidants like ascorbic acid and tocopherol in their reduced state (Dringen, 2000). A significantly low level of GSH in both liver and brain tissues during hibernation and subsequently a significant increase in its level during arousal compared to the active period was found in this study. It further declined during the post arousal phase almost to the level of GSH as it was in the active period. A decrease in GSH during hibernation might be due to its decreased biosynthesis and regeneration from GSSG, in a hypometabolic state, and low body temperature. Moreover, it has been reported about the energy-consuming GSH biosynthesis (Hermes-Lima, 2004) which might have caused a decreased level of GSH in several organs during hibernation (Orr et al., 2009). The subsequent increase in GSH level during arousal is an adaptive response to neutralize the increased production of ROS due to raised O_2_ consumption and rewarming. The increased GSSG observed in this investigation might be due to its raised production as a result of higher neutralization of ROS by GSH. It could also be due to its reduced turnover into GSH because of low glutathione reductase (GR) activity during low body temperature and hypometabolic state of hibernation. GSHeq (2GSSG+GSH) like GSH was also found significantly low during hibernation in both the tissues in spite of a significant increase in the GSSG level. It is mainly due to a significant decrease in GSH level during hibernation. It further significantly increased during the arousal phase to counteract the ROS attack due to higher oxygen consumption and rewarming. This significant increase is due to a significant increase in both GSH and GSSG levels during the arousal phase. So it indicates, though there is an adaptive increase in GSH level to counteract the increased ROS production due to increased oxygen consumption and rewarming during the arousal phase, there is also significant neutralization of ROS by GSH to produce a higher number of GSSG during increased ROS attack. It again decreased significantly during the post arousal phase to reach the level that was in the active period indicating normalcy.

In this investigation oxidative stress during the hibernation and arousal phase was found with a significant increase in lipid peroxidation, protein carbonylation, and GSSG:GSH ratio. Out of the different antioxidants studied, catalase and ascorbic acid were found upregulated during hibernation, and almost all the enzymatic and nonenzymatic antioxidants were found in a higher level of their activity during the arousal phase to counteract the effects of increased ROS following increased O_2_ consumption and rewarming. In contrast to earlier reports in different ectothermic animals, our report showing oxidative stress during hibernation and arousal evidenced from higher lipid peroxidation, protein carbonylation and GSSG:GSH ratio level and changes in both antioxidant enzymes and nonenzymatic antioxidant levels during different phases like summer active, hibernation, arousal and post arousal is unique and reported for the first time.

Hibernation in *Duttaphrynus melanostictus* characterized by hypometabolism and low body temperature during the winter season is an adaptive response towards low environmental temperature and scarcity of food materials. Increased oxidative stress markers in terms of lipid peroxidation, protein carbonylation, and GSSG and GSH were found in both liver and brain tissues during hibernation. To counteract oxidative stress augmented catalase activity and raised ascorbic acid levels were observed. A low level of GSH and a higher level of GSSG during hibernation indicate decreased synthesis of GSH and its turnover from GSSG due to low GR activity during hypometabolism and low body temperature. A decrease in uric acid level during hibernation points to its low rate of synthesis in the hypometabolic state. A comparatively higher level of ascorbic acid in brain tissue than liver tissue indicates its neuroprotective role during oxidative stress. During arousal from hibernation, there was an augmentation of both SOD and catalase activities and increased levels of nonenzymatic antioxidants like ascorbic acid, uric acid, and reduced glutathione to protect oxidative assault due to raised oxygen consumption and rewarming. Coming down of oxidative-stress markers like lipid peroxidation and protein carbonylation level to the level that was in active toads concomitantly with the decrease of enzymatic and nonenzymatic antioxidant level in both the tissues during the post-arousal phase indicates controllability of oxidative stress in hibernating toads.

## MATERIALS AND METHODS

### Animal collection and experimental conditions

Animal collection and experiments were as per the directives of the institutional animal ethics committee of Berhampur University, India, Registration number 2020/GO/Re/S/18/CPCSEA**,** and resolution number 01. Matured (4 year-old) male common Asian toads, *Duttaphrynus melanostictus* with a snout–vent length of 8.0 –8.3 cm and body weight of 39–50 ***g***, found in their natural habitat (an area protected with boundary wall surrounding abandoned houses, bushes, swampy areas and some staff quarters) located in Paralakhemundi (10° 45̓′ N, 84° 6′ E), India, were selected for this study. The ages of these animals were ascertained by skeletochronology (Sahoo and Kara, 2017). Toads of the same age with almost the same body weight and snout to vent length were selected to ensure uniformity of samples. Males were identified by observing a brick red- or orange-colored hue on the throat region and black nuptial pads on the inner sides of the first two fingers of the forelimb. Females were not chosen for this experiment due to their role in reproduction for multiplying the number of individuals in the population. In this study, 28 matured male toads were collected in four different periods from their natural habitat at the rate of seven toads (*n*=7) each time for comparison of oxidative stress parameters and antioxidant defense status among them. Morphometric parameters of common Asian toads collected during different environmental conditions from their natural habitats for comparison of oxidative stress parameters and antioxidant defense status during different phases of hibernation were shown in Table 2. While summer active toads were collected during June to August 2018, hibernating toads were collected from their burrows in loose moist loamy soil with air-filled spaces in between soil peds mostly in solitary conditions and sometimes in a group of two to five individuals during the first week of January 2019 having cement-gray color thick skin with dried mucous enveloping the entire body leaving only the nostrils exposed for breathing. Towards the last week of January 2019 (the late winter months) when atmospheric temperature raised, some toads came out from their burrows naturally and were considered as aroused toads and collected for an experiment. Likewise, towards the end of January and early February 2019, when normal movements for foraging were observed, they were considered post-arousal toads and collected for experiment.

**Table 2.**
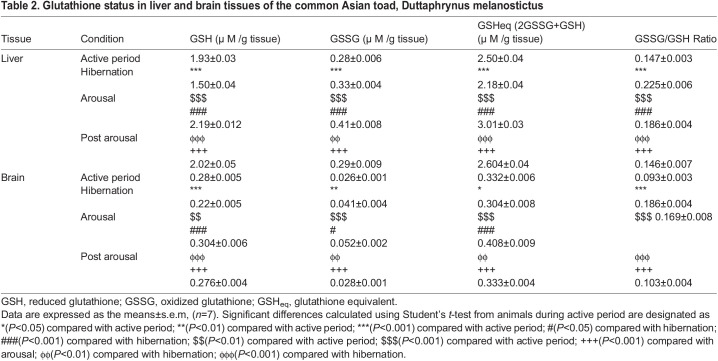
Morphometric parameters of common Asian toads collected during different environmental conditions from their natural habitats

### Tissue preparation

Four different groups (*n*=7) like summer active, hibernating, arousing, and post arousing groups of toads were collected from their natural habitat and immediately decapitated to dissect out the whole liver and brain and stored in ice-cold (2°C) amphibian Ringer's solutions. After removing adherent tissues, the organs were weighed and immediately processed for different estimations of oxidative stress parameters and antioxidants defense status.

### Lipid peroxidation assay

Lipid peroxidation (LPO) level in terms of thiobarbituric acid reactive substances (TBARS) formed was estimated by the thiobarbituric acid (TBA) test, as described by Sestini et al., (1991). Briefly, 0.5 ml 2.5% (w/v) ice-cold tissue homogenate (50 mg tissue was homogenized in a pre-cooled Teflon-glass tissue homogenizer with 2 ml of ice-cold 50 mM potassium phosphate buffer (p^H^=7.0) containing 0.5 mM EDTA (Himedia Laboratory Pvt. Ltd, India) and a few crystals of phenylmethylsulfonyl fluoride), 0.5 ml of 0.6% TBA (Sigma-Aldrich, USA) and1.5 ml of 1% orthophosphoric acid were heated in a hard glass test tube for 45 min at 95°C. A control tube containing 0.5 ml of distilled water instead of tissue homogenate was also run in parallel to the experimental tube. Both the set of test tubes were immediately cooled to room temperature under tap water, 3 mL of chloroform and 1 mL of glacial acetic acid were added to each of the test tubes and centrifuged (1000×***g***) for 10 min. Extinction of the upper phase of the biphasic supernatant containing TBARS in the experimental tube was measured at 535 nm in a UV-VIS-spectrophotometer (cystronic-119) against the control. The TBARS content in the tissue calculated using a molar extinction coefficient of 1.56×10^5^M^−1^cm^−1^ was expressed as µmol/***g*** tissue wet weight.

### Protein carbonylation assay

The carbonyl content of proteins in a sucrose soluble 2.5% (w/v) tissue homogenate [50 mg tissue was homogenized in a pre-cooled Teflon-glass tissue homogenizer with 2 ml of ice-cold 0.25 M sucrose solution followed by centrifugation at 1000×***g*** for 10 min at 4°C using cold centrifuge REMI, India to produce the supernatant as 2.5% (w/v) tissue homogenate], was estimated following the methods of Uchida and Stadtman (1993). Briefly, 0.8 ml of 0.25 M sucrose soluble tissue homogenate (2.5%w/v) and 0.8 ml of 0.1% (w/v) 2,4 dinitrophenyl hydrazine (DNPH) in 2N HCl were incubated at room temperature (25±2°C) for 1 h in the dark, and control tube was also run simultaneously along with the experimental tube with 0.8 ml of 2N HCl instead of DNPH. Protein fractions were obtained by centrifugation (1000×***g*** for 10 min) with 0.8 ml of 20% trichloroacetic acid (TCA). They were washed with (1:1 V/V) ethanol and ethyl acetate mixture and then dissolved in 2 ml of 8 M guanidine hydrochloride (Himedia Laboratory Pvt. Ltd, India) prepared in 133 mM Tris buffer (P^H^=7.2) containing 13 mM EDTA. The extinction of the experimental sample was measured at 365 nm against the control. The carbonyl content was expressed as nanomoles of DNPH incorporated per mg protein, based on the molar extinction coefficient of 22×10^3^ M^−1^cm^−1^. The protein content of the tissue homogenate was estimated following the method of Lowry et al. (1951) using bovine serum albumin as standard.

### Assay of SOD activity

Cytosolic Cu and Zn-containing forms of SOD (E.C.1.15.1.1) activity in liver and brain tissues were estimated following the method of Das et al., (2000). In this method, superoxide radicals are generated by photoreduction of riboflavin (Beyer and Fridovich, 1987) and its detection by nitrite formation from hydroxylamine hydrochloride as described by Elstner and Heupel (1976) and modified by Pattichis et al., (1994) using Greiss reagent. 2 ml of 2.5% (w/v) tissue homogenate prepared in ice-cold 50 mM phosphate buffer (p^H^=7) containing 1 mM EDTA and few crystals of phenylmethylsulfonyl fluoride was centrifuged at 10,000×***g*** for 20 min at 4°C by cold centrifuge, REMI, India. The supernatant (1 ml) was allowed to pass through a 5 ml column of Sephadex G-25(Sigma-Aldrich, USA), to collect the elute for the essay of SOD. A cocktail of 1.4 ml prepared by adding 1.11 ml of 50 mM phosphate buffer (p^H^=7), 0.075 ml of 20 Mm L-methionine, 0.04 ml of 1% (v/v) triton ×100, 0.075 ml of 10 mM hydroxylamine hydrochloride (HAC) (Himedia Laboratory Pvt. Ltd, India) and 0.1 ml of 78.125 mM EDTA was added with 0.1 ml of tissue elute and 0.1 ml of 40 µM riboflavin in the experimental tube. A blank prepared with riboflavin and control without tissue elute were also run simultaneously with each test. All the test tubes were exposed to two 20 watt fluorescent lamps for 10 min followed by the addition of 1mL of Griess reagent and measurement of extinction of both control and experimental tubes at 543 nm in a spectrophotometer against the blank. SOD activity was expressed as units/mg/protein where 1 unit of enzyme activity=(V_0_/V) −1 in which V_0_ and V represent the extinction of control and experimental tubes respectively.

### Assay of catalase activity

Catalase (E.C 1.11.1.6) activity in the supernatant obtained by centrifugation (10,000×***g*** for 20 min at 4°C by cold centrifuge, REMI, India) of 2.5% (w/v) tissue homogenate in ice-cold 50 mM phosphate buffer (P^H^=7.4) containing 1 mM EDTA and few crystals of phenylmethylsulfonyl fluoride was used following the decrease in absorbance of H_2_O_2_ at 240 nm (Aebi, 1974). Briefly, 0.1 ml of supernatant was added to 2.9 ml of 50 mM phosphate buffer (P^H^=7.4) containing 12 mM H_2_O_2_to start enzymatic reaction at room temperature, and the decrease in extinction was recorded immediately at 240 nm at a 1 min interval for three minutes in a UV-visible spectrophotometer against the blank having the only 3 ml of 50 mM phosphate buffer (P^H^=7.4). A decrease in the concentration of H_2_O_2_ (µmol/ml) per minute by the action of catalase present in 0.1 ml of supernatant was calculated from the extinction coefficient of H_2_O_2_, i.e. 43.6×M^−1^cm^−1^, and the catalase activity was expressed as µM H_2_O_2_ consumed/min/mg protein.

### Ascorbic acid

Ascorbic acid estimation was done by using a deproteinized supernatant obtained from centrifugation (1000×***g*** for 10 min) of 2.5% (w/v) tissue homogenate prepared from 6% ice-cold TCA, following Roe (1954) method. Ascorbic acid present in deproteinized tissue extract was oxidized to dehydroascorbic acid (DHAA) using bromine water, which transformed irreversibly to 2, 3-diketogulonic acid (DKA). The DKA coupled with 2, 4-dinitrophenyl hydrazine (DNPH) to form a colored product with H_2_SO_4_. Briefly to 2.0 ml of tissue extract, 0.5 ml of 2, 4-dinitrophenyl hydrazine-thiourea reagent was added and then incubated a 57°C for 45 min in a temperature-controlled water bath. After cooling to room temperature, 5 ml of 85% H_2_SO_4_ was added dropwise and extinction of the colored product that formed after 30 min of incubation was measured at 530 nm against the blank containing 2 ml of 6% TCA instead of tissue extract. The ascorbic acid content in the tissue extract was determined from the standard curve of ascorbic acid and expressed as µg ascorbic acid/***g*** wet tissue.

### Uric acid

The uric acid content in the deproteinized supernatant obtained by centrifugation (1000×***g*** for 10 min) of 0.5 ml of 2.5% (w/v) tissue homogenate prepared in ice-cold 50 mM phosphate buffer (pH=7.0), 4 ml of N/23 H_2_SO_4,_ and 0.5 ml of 5.6% sodium tungstate was estimated as described by Buchanan et al., (1965). Extinction of the light blue-colored product formed by adding 0.2 ml of phosphotungstic acid reagent and 1 ml of 0.6N NaOH to 3 ml of deproteinized supernatant was measured at 720 nm and the result was obtained from the standard curve of uric acid. The uric acid content was expressed as µg uric acid/***g*** wet tissue.

### Total and oxidized glutathione

Total glutathione equivalents (GSH_eq_) consisting of both GSH and GSSG were measured following the method of Griffith (1980). A protein-free supernatant was obtained by centrifugation (10,000×***g*** for 15 min at 2°C) of the tissue homogenate (1:5w/v) in ice-cold (2°C) sulfosalicylic acid, was divided into two parts. One part was used to measure GSH_eq_ by observing the rate of reduction of DTNB at 412 nm containing 0.2 mM NADPH, 0.6 mM DTNB, 5 mM EDTA, 125 mM sodium phosphate buffer (pH=7.5), and tissue extract in a final volume of 1mL. To ensure the rate of reaction was zero, the reaction was started by adding GR (0.5 U). The rate of reaction is proportional to the concentration of GSH_eq_ and was compared with the standard curve of GSH (0-6 µM). Another part of the protein-free supernatant was treated with 170 mM 2-vinyl pyridine for 1 h to derivatize GSH. The rest of the GSSG was measured, and the total GSH was calculated from the equation GSH_eq_=GSH+2GSSG, and the result was expressed as µmol/***g*** tissue wet weight. The levels of GSH (GSH=GSH_eq_-2GSSG) and percent oxidized GSH (GSSG/GSH) were also calculated.

GR and DTNB were purchased from Sigma-Aldrich, USA; NADPH, GSH, GSSG, 2-vinyl pyridine, guanidine hydrochloride, EDTA were obtained from HiMedia Laboratories Pvt. Ltd., India.

### Statistical analysis

Data were expressed as means±s.e.m (*n*=7). One-way ANOVA with post hoc (DUNCAN multiple range tests) was made using IBM, SPSS-25.0. Additionally, the Student's *t*-test was used to compare the mean values of two different groups at a time. A *P*-value less than 0.05 was considered statistically significant.
